# Training Service Users in the Use of Telehealth: Scoping Review

**DOI:** 10.2196/57586

**Published:** 2024-07-31

**Authors:** Emer Galvin, Shane Desselle, Blánaid Gavin, Fiona McNicholas, Shane Cullinan, John Hayden

**Affiliations:** 1 School of Pharmacy and Biomolecular Sciences Royal College of Surgeons in Ireland Dublin Ireland; 2 Touro University California California, CA United States; 3 School of Medicine University College Dublin Dublin Ireland; 4 Children’s Health Ireland Dublin Ireland; 5 Lucena Child and Adolescent Mental Health Service (CAMHS) Dublin Ireland

**Keywords:** telehealth, video consultations, training, education, older adults, digital divide, digital literacy, review, scoping review, modality of care, training service, user, users, older adult, gerontology, geriatric, geriatrics, caregiver, caregivers, consultation, consultations, health care professional, health care professionals, PRISMA-ScR, Preferred Reporting Items for Systematic Reviews and Meta-Analyses Extension for Scoping Reviews, data extraction, phone, phones, telemonitoring

## Abstract

**Background:**

The use of telehealth has rapidly increased, yet some populations may be disproportionally excluded from accessing and using this modality of care. Training service users in telehealth may increase accessibility for certain groups. The extent and nature of these training activities have not been explored.

**Objective:**

The objective of this scoping review is to identify and describe activities for training service users in the use of telehealth.

**Methods:**

Five databases (MEDLINE [via PubMed], Embase, CINAHL, PsycINFO, and Web of Science) were searched in June 2023. Studies that described activities to train service users in the use of synchronous telehealth consultations were eligible for inclusion. Studies that focused on health care professional education were excluded. Papers were limited to those published in the English language. The review followed the Joanna Briggs Institute guidelines for scoping reviews and was reported in line with the PRISMA-ScR (Preferred Reporting Items for Systematic Reviews and Meta-Analyses Extension for Scoping Reviews) guidelines. Titles and abstracts were screened by 1 reviewer (EG). Full texts were screened by 2 reviewers (EG and JH or SC). Data extraction was guided by the research question.

**Results:**

The search identified 8087 unique publications. In total, 13 studies met the inclusion criteria. Telehealth training was commonly described as once-off preparatory phone calls to service users before a telehealth visit, facilitated primarily by student volunteers, and accompanied by written instructions. The training content included guidance on how to download and install software, troubleshoot technical issues, and adjust device settings. Older adults were the most common target population for the training. All but 1 of the studies were conducted during the COVID-19 pandemic. Overall, training was feasible and well-received by service users, and studies mostly reported increased rates of video visits following training. There was limited and mixed evidence that training improved participants’ competency with telehealth.

**Conclusions:**

The review mapped the literature on training activities for service users in telehealth. The common features of telehealth training for service users included once-off preparatory phone calls on the technical elements of telehealth, targeted at older adults. Key issues for consideration include the need for co-designed training and improving the broader digital skills of service users. There is a need for further studies to evaluate the outcomes of telehealth training activities in geographically diverse areas.

## Introduction

The use of telehealth, defined as live audio and video consultations between service users and health care professionals, has increased exponentially since the onset of the COVID-19 pandemic [1]. The presence of physical distancing restrictions and stay-at-home orders necessitated this form of health care delivery [2]. The use of telehealth has prevailed beyond the pandemic, due to its benefits to service users and clinicians alike. These benefits include timely access to care, removal of logistical barriers, and convenience [3]. While these benefits are lauded, many underserved populations, such as those of low income, still face barriers to accessing telehealth [4,5]*.* One reason for this disparity in access may be because of the presence of the “digital divide.”

The digital divide has been recognized as an important social determinant of health [6] and is recognized as having 3 elements [7]. These are (1) disparities in access to technologies, (2) disparities in skills to be able to use these technologies efficiently, and (3) disparities in people’s ability to use these technologies to achieve outcomes to improve their lifestyle [7,8]. While device ownership and internet access are increasing worldwide, gaps in digital skills and competencies are still prevalent [9]. In the United Kingdom, 21% of the population lack basic digital skills [10]. These inequalities in digital skills are recognized as contributing to disparities in telehealth use [5,9,11,12].

It is now critical that there are programs to improve the digital literacy of service users so that they have the confidence and skills to use, and benefit from, telehealth [13]. Research on training health care professionals in telehealth has grown, with recent studies identifying the most necessary competencies and approaches for training professionals of various health care disciplines [13,14]. There are now calls for interventions to improve the digital skills of service users who experience barriers to telehealth [12]. Specifically, researchers have explicitly called for training and education on the use of telehealth to increase access for underserved populations [15,16]. This training could have the potential to narrow the widening disparities in telehealth access yet remains an understudied area of research.

This review aimed to examine the extent to which training and education activities for service users in telehealth are reported in the literature, in addition to summarizing the findings of this research. The review also aimed to identify gaps in the literature and determine future research needs.

## Methods

### Approach

A scoping review was identified as the most relevant method to answer the review question [17], as the research question was exploratory and aimed to map the breadth and heterogeneity of the literature. This review design was chosen to provide an initial idea of the size and nature of the available research and to identify gaps in the existing literature [17,18].

The Joanna Briggs Institute guidelines for scoping reviews [19] were followed and the review was reported in line with the PRISMA-ScR (Preferred Reporting Items for Systematic Reviews and Meta-Analyses Extension for Scoping Reviews) checklist (Multimedia Appendix 1 [20]). The 5-step scoping review methodologies by Arksey and O’Malley [18] and Levac et al [21] were followed. The five steps were (1) to identify the research question; (2) to identify relevant studies; (3) to select studies; (4) to chart the data; and (5) to collate, summarize, and report results. This scoping review was not registered. The protocol was published on the Open Science Framework website [22].

### Step 1: Identifying the Research Question

The scoping review aimed to answer the following question: What is the nature and extent of the literature related to training and education activities for service users in telehealth? The review aimed to provide a comprehensive overview of the breadth and heterogeneity of published research on the provision of training for service users in telehealth. The scoping review also aimed to identify gaps in the literature, limitations of the research, and directions for future research. The specific aims were to (1) summarize the research on training and education activities for service users in telehealth and (2) summarize the content and main features of these activities.

### Step 2: Data Source and Search Strategy

The search strategy aimed to locate published studies on the topic of telehealth training for service users. Initial searches were conducted on PubMed and Google Scholar to identify potentially relevant studies. The terms and keywords of these studies were used to create the search strategies. The search strategies were piloted to identify the most appropriate search terms and subsequently were adapted to the parameters of each database. The lead author (EG) conducted the searches in June 2023 of the following databases: MEDLINE (via PubMed), Embase, CINAHL, PsycINFO, and Web of Science. The full search strategies can be seen in Multimedia Appendix 2. Only studies published in English were included. Backward and forward citation tracking was conducted by the lead author (EG).

### Step 3: Eligibility Criteria and Screening Process

EndNote X9 (Clarivate) was used to screen and organize the records. The following eligibility criteria were applied when screening the studies.

#### Participants

Adult populations, including parents and caregivers, were eligible for inclusion. Child populations (younger than 18 years) were excluded, due to an absence of relevant studies found in the initial searches.

#### Concept

Telehealth was defined as the provision of health care services to service users by health care professionals, through live, synchronous audio and video consultations. Studies that focused on training adult service users on how to use telehealth, including how to set up telehealth, were included. Studies that focused on how to set up telemonitoring devices (eg, heart monitors and spirometers) only were excluded. Studies that focused on training users to use patient portals, unless there was a telehealth consultation component, were excluded. Studies that focused on health care professional education only were excluded.

#### Context

Studies of service users in any setting were eligible for inclusion.

#### Types of Sources

Studies of all research designs, including quantitative, qualitative, and mixed methods studies, were eligible for inclusion. Systematic reviews and literature reviews were excluded. Text and opinion papers were eligible for inclusion if they included adequate information on education and training activities. Conference abstracts were excluded, as they did not provide sufficient information required for this scoping review.

#### Study or Source of Evidence Selection

The lead author (EG) screened the titles and abstracts of all publications identified in the database searches. Two reviewers (EG and JH or SC) screened the full texts of the papers for inclusion. Any disagreements were resolved by discussion. Reasons for exclusion of studies at full-text screening were recorded and reported. The results of the search and the study inclusion process were presented in a PRISMA-ScR flow diagram [19].

### Step 4: Data Extraction

A data extraction tool was developed using Microsoft Excel. The extracted data included author, year of publication, aim of study, sample, description of training, outcome measures, and key findings. Papers were excluded at this stage if the reviewers agreed there was insufficient data on the topic. The lead author (EG) conducted data extraction of all included papers.

### Step 5: Data Synthesis

As recommended by Arksey and O’Malley [18], the quality of the evidence was not assessed. A descriptive narrative synthesis and numerical summaries were conducted to present the findings.

## Results

### Study Characteristics

**Figure 1:**
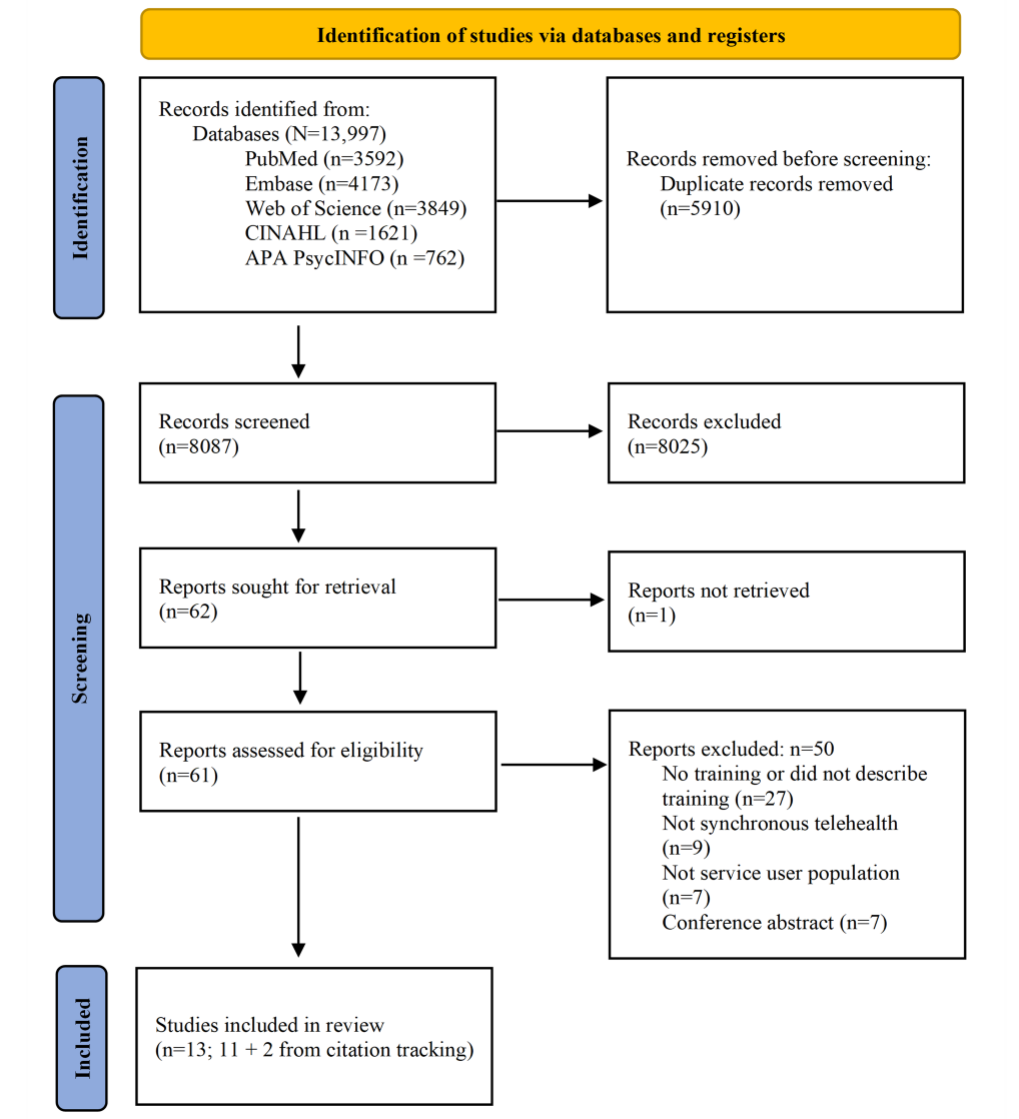
PRISMA (Preferred Reporting Items for Systematic Reviews and Meta-Analyses) flow diagram for scoping reviews.

The database searches yielded 13,997 records. After removing duplicates and applying the eligibility criteria, 13 papers were included in the review [23-35] (see Figure 1 for the PRISMA [Preferred Reporting Items for Systematic Reviews and Meta-Analyses] flow diagram). All 13 studies were published between 2020 and 2023. All studies were conducted in the United States and were published as journal papers. Study designs included uncontrolled preintervention and postintervention studies (n=4, 31%), postintervention studies (n=4, 31%), brief communications (n=4, 31%), and a retrospective cohort study (n=1, 8%). Studies were conducted in academic medical centers (n=5, 39%), geriatrics clinics (n=3, 23%), community settings (n=3, 23%), a specialty care clinic (n=1, 8%), and a federally qualified health center (n=1, 8%). A total of 12 (92%) studies were conducted during the COVID-19 pandemic, in the context of the rapid implementation of telehealth at this time. In total, 9 (69%) of the training programs were evaluated empirically, using mixed methods (n=5, 39%) and quantitative methods (n=4, 31%). The remaining 4 (31%) studies described training activities in “brief communication” style papers detailing their institution’s wider move to telehealth during the pandemic. The full characteristics of the included studies can be seen in Multimedia Appendix 3 [23-35].

### Service User and Trainer Characteristics

Of the 13 studies, the target populations included older adults (n=7, 54%), patients in hospital outpatient settings (age unspecified; n=4, 31%), and adult patients (n=2, 15%). In the 6 studies that reported the demographic information of service users, the majority of participants were female (55%-79%) [23,24,26,29,31,35] and predominantly White (42%-79%) [24,26,29,31,35]. In 1 of the 6 studies, the population was predominantly Black (47%) [23]. A total of 3 (23%) of the 13 studies asked participants about access to technological devices and the internet. Jezewski et al [29] reported that 93% of participants had access to a telehealth-compatible device. Antonio et al [23] reported that 60% of participants had access to a laptop and 99% had access to a cell phone. Hawley et al [27] reported that 64% of participants had access to both the internet and an internet-compatible device with a camera.

The training was delivered primarily by medical or health professions student volunteers (n=7, 54%), followed by hospital staff (n=3, 23%), volunteer trainers (n=1, 8%), graduate students (n=1, 8%), and a member of the research team (n=1, 8%). A total of 4 (31%) studies described the training the trainers undertook, and 5 (39%) studies described that the trainers had materials to assist them such as scripts [24,26] and instructions [23,30,32]. Two training initiatives were facilitated by partnerships with community organizations [29,31].

### Training Format, Delivery, and Duration

One-on-one phone calls prior to the telehealth visit were the most commonly used training formats (n=9, 69%). Other training formats included a Microsoft PowerPoint presentation (n=1, 8%), a one-on-one video call (n=1, 8%), a prerecorded video (n=1, 8%), and web-based modules (n=1, 8%). Many studies (n=9, 69%) also provided written instructional documents to participants, primarily on how to download telehealth applications. In addition, 1 (8%) study also included a video including actors that simulated a telehealth consultation, as part of the web-based modules [35]. Phone was the most commonly used delivery modality (n=9, 69%), followed by web-based (including modules, video calls, and prerecorded video; n=3, 23%), and a mix of in-person and paper-based (n=1, 8%). Studies additionally used email (n=5, 39%) and SMS text messaging (n=3, 23%) to communicate with and send materials and web links to participants.

**Figure 2:**
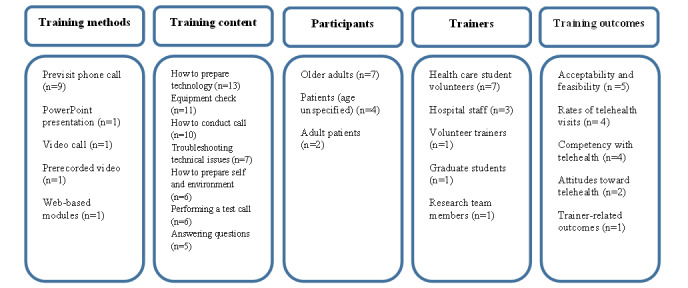
Summary of training characteristics.

In terms of duration, most of the studies were once-off phone calls (n=8, 62%). The in-person PowerPoint presentation was 20 minutes long but moved to a self-paced paper-based presentation because of COVID-19 restrictions [29]. The web-based modules described by Taylor et al [35] were also self-paced. One training program lasted 1 to 2 hours over 7 sessions, across 2 months in total [31]. Pichan et al [32] described conducting 3 phone calls over the course of 1 week. The prerecorded video described by Spindler et al [34] was less than 2 minutes in duration. A summary of training characteristics is presented in Figure 2.

### Training Objectives and Content

All studies aimed to train service users on how to use telehealth, primarily to prepare them for upcoming video visits. Additional training-related aims were to reduce the cognitive load demands of telehealth [23], develop health professions students’ communication skills [25], and improve the broader digital literacy of service users [31]. The content of the training activities primarily focused on helping service users set up for a video call. All studies provided training on how to prepare the technology including downloading video applications. A majority of 11 (85%) studies described checking with service users that they had the necessary equipment to conduct video calls. A total of 10 (77%) studies described providing guidance on how to conduct a video telehealth call, while 6 (46%) studies described guiding service users on how to prepare themselves or their environment for a video telehealth call. In total, 7 (54%) studies described providing troubleshooting training for technical issues with participants, while 6 (46%) studies described performing a test video call with the service users. A total of 5 (39%) studies described answering service user queries about telehealth.

In total, 4 (31%) studies described explaining to service users what telehealth is, with 2 (15%) studies reported describing to service users how telehealth can be used. A total of 3 (23%) studies provided guidance on how to ensure safety and privacy while using telehealth, with only 1 (8%) study describing the limitations of telehealth [35]. Finally, only 1 (8%) study focused on teaching service users about elements of care during the telehealth visit, including care planning in telehealth, forming a therapeutic relationship, and team-based care in telehealth [35].

### Development of Training

In total, 7 (54%) studies reported details on how the training activities were developed. A total of 2 studies described developing the training using the theoretical underpinnings of cognitive load theory [23] and provider telehealth training [35], respectively. A total of 2 studies [29,31] described partnering with community organizations to develop training programs. Hawley et al [27] described categorizing patients into 4 phenotypes based on their interest and capability to complete a home telehealth visit and subsequently creating training to overcome patient-perceived barriers. Pichan et al [32] described that medical students developed the training program, and Gulati et al [25] reported that the geriatrics faculty lead provided guidance on the design of the program.

### Reasons for Not Participating or Dropping Out

A total of 4 (31%) studies described reasons why people did not accept training or dropped out of the training program. Reasons for declining assistance or training included lack of interest in telehealth [23,24], did not feel they needed assistance [24], had already canceled their appointment [24], lack of device [23], and having existing telehealth experience [23]. Reasons for dropping out of training programs included because of health issues [31], schedule conflicts [31], participants not responding [31], and internet problems [31]. Taylor et al [35] reported that lower median household income was significantly associated with lower completion of the training program.

### Evaluation of Training Programs

#### Overview

Studies used a number of methods to examine the outcomes of the training programs. A total of 5 (39%) studies examined qualitative outcomes and 9 (69%) studies examined quantitative outcomes. Studies examined the impact of training on rates of telehealth video visits, telehealth competency and confidence, attitudes toward telehealth, acceptability and feasibility of training, and trainer-related outcomes.

#### Rates of Telehealth Video Visits

In total, 4 (31%) studies examined the impact of training on rates of telehealth video visits [23,24,26,32]. Gusdorf et al [26] reported that a training call significantly increased the likelihood of a successful video visit. Chu et al [24] reported that the majority of participants who received training (76.5%) were successfully video enabled and that those who declined training had the highest rate of video visit cancellation. Pichan et al [32] reported that rates of video visits increased from before, to after, participating in training. Finally, Antonio et al [23] reported that there was no difference in video visit rates between intervention and nonintervention participants following training and that 40% of intervention participants who were scheduled for video visits ultimately had a phone visit.

#### Competency and Confidence With Telehealth

Changes in competency and confidence with telehealth were examined in 4 (31%) studies [23,29,31,35]. Taylor et al [35] reported significant improvements in perceived telehealth competency following training. Neumann et al [31] found a significant increase in the mean perceived confidence level for engaging in video visits following training. Antonio et al [23] found no significant differences in self-efficacy or perceived difficulty in using video visits between intervention and nonintervention participants following training. Jezewski et al [29] reported that 36% of participants were familiar with telehealth before training, and after training, 70% of participants understood how to access telehealth. However, 21% of participants reported wanting more information about telehealth after training.

#### Attitudes Toward Telehealth

A total of 2 (15%) studies examined the impact of training on telehealth attitudes [23,29]. Jezewski et al [29] reported that 39% of participants would use telehealth following training. The authors did not report what this value was before training. In Antonio et al [23], intervention participants were significantly less satisfied with their video visit experience than nonintervention participants following training.

#### Acceptability and Feasibility of Training

A total of 5 (39%) studies examined the acceptability and feasibility of training [23,27,31,32,35]. Participants in a number of studies appreciated the human, one-on-one elements of training. Antonio et al [23] reported that participants appreciated the communication skills of their trainer and the relationship and rapport they had with the trainer. Similarly, Neumann et al [31] reported that participants valued the patience and reinforcement from the trainers and described having personal relationships with them. This view was mirrored by participants in Pichan et al [32], who appreciated the time the trainers put into helping them.

Certain elements of the training were valued and helpful to participants. Antonio et al [23] reported that participants appreciated the step-by-step guidance offered in the training sessions. Similarly, participants in Antonio et al [23] valued the structure of the training, such as the repetition and self-pacing. Participants in Neumann et al [31] appreciated having access to the devices and training booklets. Having a partnership with a trusted community organization was also appreciated by participants [31]. Pichan et al [32] solicited feedback from providers, who expressed positive views, highlighting that the training program increased their video visits and that patients felt empowered to learn new skills and appreciated the training sessions. Similarly, Antonio et al [23] reported that participants valued being able to access health services and they described having a sense of purpose and control, and not having to rely on family members to help. Hawley et al [27] reported that participants found the training and instructions helpful.

Of the 13 included studies, 4 (31%) studies reported service users’ and trainers’ challenges relating to the telehealth training programs [23,31,32,35]. The challenges described by learners participating in telehealth training included the accessibility of training [35], concerns about the privacy of the website hosting the training [35], technological security [31], pandemic (isolation) [31], health status and care change [31], and working with older devices [23,32]. Other challenges included adjusting sound and visual settings on devices [23,32], internet access problems [23], switching between applications on small devices [23], people in the background impacting training [23], and patients having to log in to their patient portal to access the telehealth video platform [23]. Taylor et al [35] revised their training modules based on participant feedback. From the clinician’s perspective, the challenges included the time-consuming task of going through their patient list and compiling the patient’s information for their volunteer trainer [32].

#### Trainer-Related Outcomes

Gulati et al [25] examined outcomes relating to the trainer who provided the telehealth training calls. They reported that health professions students experienced improvements in their health communication skills.

## Discussion

### Principal Findings

This scoping review identified 13 studies that described training service users in the use of telehealth. All of the studies were conducted in the United States. The target training groups were primarily older people and patients in academic medical centers. The most commonly reported training method involved a preparatory phone call before a telehealth visit conducted by student volunteers, accompanied by written instructions. The content of training activities was similar across studies, primarily focused on assisting people to download and set up applications and devices. All but 1 of the studies were conducted during the COVID-19 pandemic, with training initiatives conducted to facilitate the rapid move to telehealth during this time.

The studies were primarily quantitative and mixed methods. Training initiatives were evaluated using a variety of methods, including examining video visit rates, conducting pretraining and posttraining surveys, and eliciting qualitative feedback. Some studies reported that telehealth training increased the amount of people participating in video visits. The evidence was limited and mixed about whether telehealth training increases service users’ perceived competency with telehealth. The available evidence suggested that training did not improve participants’ attitudes toward telehealth. Participants discussed the positive elements and challenges of training programs.

### Comparison With Prior Work

To the authors’ knowledge, this is the first scoping review to map the literature on training activities for service users in the use of synchronous telehealth. A previous review by Grossman et al [36] explored the impact of interventions to increase patient portal use. They found that individually focused interventions and technical training and assistance had the best evidence for increasing portal use. In our review, individually focused training and technical assistance were the most common elements of telehealth training. However, there was limited evidence on the effectiveness of telehealth training to improve telehealth competency. In addition, some studies reported increased rates of video visits, but it is difficult to ascertain if training alone was responsible for these increases, due to the concurrent increase in the use of video visits during the COVID-19 pandemic.

Much of the previous research on telehealth training has focused on training health care professionals and students on the use of telehealth. Studies have aimed to identify what competencies are needed for health care professionals to be proficient in telehealth. For example, Davies et al [37] conducted a Delphi study to develop a framework for physiotherapists to deliver quality care via telehealth. The framework consisted of 60 capabilities across 7 domains: compliance, patient privacy and confidentiality, patient safety, technology skills, telehealth delivery, assessment and diagnosis, and care planning and management. In this review, technology skills were the primary domain covered in the included studies. The service user training activities focused primarily on the technical elements of setting up and using telehealth. Much fewer studies focused on training in nontechnical elements such as preparing oneself for a visit, getting the most out of a visit, building a therapeutic relationship, and information about the privacy and security of telehealth. Many of the studies were conducted in the context of the COVID-19 pandemic where the priority was moving service users onto telehealth quickly, so it is likely that focusing on downloading and installing the telehealth applications was the priority of institutions.

### Implications for Practice and Research

The findings of this review highlight lessons for future training activities for service users in the use of telehealth. In total, 2 studies described partnering with community organizations to provide telehealth training to service users. Partnering with a trusted community organization was appreciated by service users [31] and may be a potential method for facilitating telehealth training. One limitation of the included studies was that there may be a geographical bias toward participants recruited from universities, academic medical centers, and metropolitan towns and cities [32,35]. Partnering with a community organization could increase reach to diverse geographical areas and ensure people are trained in other digital health skills (eg, using patient portals and finding health information on the internet), to improve the broader digital literacy of service users.

As described in 1 of the included studies [35], consulting with service users to develop the training could be a promising method to ensure that telehealth training meets their needs. Specifically, older adults in this study made recommendations to make the training modules more accessible including changes in speech, format, and language use [35]. Considering the accessibility of training for different groups is also important. For example, using closed captioning of videos, or providing word documents that can be read by screen readers, would help to improve the accessibility of training and ensure that people with disabilities are not excluded. Finally, in another study [24], service users declined assistance as they did not have a device available for video visits. To overcome this, Neumann et al [31] gave refurbished iPads to participants. Offering devices to service users or providing the option of telephone visits would ensure that those who do not have access to devices are not excluded from telehealth training or visits.

A number of gaps were identified in the included studies that could be addressed with further research. First, all studies were conducted in the United States. This presents an opportunity for further research to be conducted in more diverse geographical contexts, particularly in low- and middle-income countries where telehealth implementation is not as widespread [38]. Second, the description of training in some of the studies was vague, particularly in the studies that discussed training within their institution’s wider transition to telehealth during the pandemic. Future studies should aim to provide more detail about who conducted the training, how it was developed, and what topics were covered. Making the training materials publicly available would be useful for institutions and organizations developing telehealth training programs. In addition, studies did not report on the costs needed to implement the training activities. This is important information for institutions evaluating the feasibility and effectiveness of an intervention, so future research should report on the cost-effectiveness of training initiatives.

One of the main findings was that there was mixed evidence that training improved service users’ perceived competency with telehealth. A total of 2 studies reported significant improvements in perceived telehealth competency and confidence from before, to after, training. However, it is important to note that these studies did not employ a non-training control group. There is a need to conduct further quantitative and qualitative evaluations of training initiatives. Understanding the effectiveness, acceptability, and feasibility of different training initiatives would help to inform best practices for training service users in telehealth. In addition, future research should use validated measures to examine changes in telehealth competency. Furthermore, examining the effectiveness of human-led training compared to self-directed training could be a promising avenue of research, given the anecdotal appreciation of human assistance in the included studies.

Research on the impact of training activities outside the context of the COVID-19 pandemic is also needed. All but 1 of the studies were conducted in response to the need for telehealth implementation during this time, which may have positively impacted participants’ interest in partaking in training. In addition, some studies reported increased rates of video visits following training, which may have been confounded by the increased availability of telehealth during this time. Research is needed to investigate if these effects are observed outside the pandemic context. Finally, many of the training activities lacked an educational or theoretically informed basis, so future research should attempt to use theory- and literature-informed training, perhaps consulting the body of research on training health care providers on the use of telehealth.

### Strengths and Limitations

A key strength of this review is that it helps to address a gap in the literature of a pertinent research area. The review aimed to describe the existing literature and identify differences and commonalities between training activities. A further strength of the review is that it is reported in line with evidence-based criteria for conducting scoping reviews developed by the Joanna Briggs Institute [19] and the guidelines for scoping reviews described in the PRISMA-ScR checklist [20]. Finally, a comprehensive search of 5 major academic databases was conducted, in addition to backward and forward citation tracking.

Given the nature of scoping reviews, we did not conduct a critical appraisal of the studies included in the review. With the various terms used to describe telehealth, we may have missed some eligible studies. Another limitation of the review is that we did not use other avenues for searching papers such as gray literature searches and contacting key authors in the area. Finally, with the recent, increased use of telehealth, it is likely that further research on this topic will be published in the coming years. There will be a need for an update of this scoping review, particularly to identify studies conducted beyond the context of the pandemic.

### Conclusions

This review aimed to map the literature on training activities for service users in telehealth. The common features of training initiatives included a preparatory phone call, volunteer trainers, and the provision of training on how to download and set up telehealth applications. The target populations consisted primarily of older adults. There was limited and mixed evidence that training improved service users’ perceived competency with telehealth. Future research should focus on empirically evaluating training activities in geographically diverse settings. The review highlights the need for co-designed training and initiatives to improve the broader digital literacy of service users.

## Appendix

Multimedia Appendix 1 PRISMA-ScR (Preferred Reporting Items for Systematic Reviews and Meta-Analyses Extension for Scoping Reviews) checklist.

Multimedia Appendix 2 Search strategy.

Multimedia Appendix 3 Characteristics of included studies.

## Abbreviations

PRISMA: Preferred Reporting Items for Systematic Reviews and Meta-Analyses
PRISMA-ScR: Preferred Reporting Items for Systematic Reviews and Meta-Analyses Extension for Scoping Reviews
