# Supplementary material for: Training Service Users in the Use of Telehealth: Scoping Review
Source: J Med Internet Res. 2024 Jul 31;26:e57586. doi: 10.2196/57586 (PMC11325118; doi:10.2196/57586)
Supplement: Multimedia Appendix 2 [file jmir_v26i1e57586_app2.docx]

|  | **PubMed** |
| --- | --- |
| 1 | telemedicine[Title/Abstract] OR telehealth [Title/Abstract] |
| 2 | education[Title/Abstract] OR training[Title/Abstract] OR course[Title/Abstract] OR module[Title/Abstract] OR skill*[Title/Abstract] |
| 3 | (patient[Title/Abstract] OR user[Title/Abstract] OR client[Title/Abstract] OR senior*[Title/Abstract] OR adult*[Title/Abstract] |
| 4 | 1 AND 2 AND 3 |
| 5 | English Language |

|  | **Embase** |
| --- | --- |
| 1 | ('telemedicine':ab,ti OR 'telehealth':ab,ti) |
| 2 | (education:ab,ti OR training:ab,ti OR course:ab,ti OR module:ab,ti OR skill*:ab,ti) |
| 3 | (patient:ab,ti OR user:ab,ti OR client:ab,ti OR senior*:ab,ti OR adult*:ab,ti) |
| 4 | 1 AND 2 AND 3 |
| 5 | English language |
| 6 | EMBASE only (Not Medline) |

|  | **Web of Science** |
| --- | --- |
| 1 | TI = ("telemedicine" OR "telehealth" ) AND (education OR training OR course OR module OR skill*) AND (patient OR user OR client OR senior* OR adult*) |
| 2 | AB = ("telemedicine" OR "telehealth" ) AND (education OR training OR course OR module OR skill*) AND (patient OR user OR client OR senior* OR adult*) |
| 3 | 1 OR 2 |
| 4 | English |

|  | **CINAHL** |
| --- | --- |
| 1 | TI ( ("telemedicine" OR "telehealth" ) AND (education OR training OR course OR module OR skill*) AND (patient OR user OR client OR senior* OR adult*) ) |
| 2 | AB ( ("telemedicine" OR "telehealth") AND (education OR training OR course OR module OR skill*) AND (patient OR user OR client OR senior* OR adult*) ) |
| 3 | 1 OR 2 |
| 4 | English |

|  | **APA PsycInfo** |
| --- | --- |
| 1 | TI ( ("telemedicine" OR "telehealth" ) AND (education OR training OR course OR module OR skill*) AND (patient OR user OR client OR senior* OR adult*) ) |
| 2 | AB ( ("telemedicine" OR "telehealth") AND (education OR training OR course OR module OR skill*) AND (patient OR user OR client OR senior* OR adult*) ) |
| 3 | 1 OR 2 |
| 4 | English |
