# Supplementary material for: Training Service Users in the Use of Telehealth: Scoping Review
Source: J Med Internet Res. 2024 Jul 31;26:e57586. doi: 10.2196/57586 (PMC11325118; doi:10.2196/57586)
Supplement: Multimedia Appendix 3 [file jmir_v26i1e57586_app3.docx]

| **Authors, article type** | **Study design, population, setting** | **Aim of training** | **Training characteristics** | **Training evaluation measures** | **Key findings** |
| --- | --- | --- | --- | --- | --- |
| **Authors:**  Antonio et al. 2023  **Article type:**  Empirical | **Design:** Pre-post mixed methods pilot intervention study.  **Population and setting:** 239 adult patients, Federally Qualified Health Centre, USA. | To reduce the cognitive load demands required to learn and use telehealth. | **Delivery mode:** Phone. Test video call. PDF document.  **Frequency:** Single, 10 – 30 minute session.  **Trainer:** Graduate student research assistant.  ***N* participants received training:**  34. | Intervention recruitment tracking (completed by helpers).  Structured observations of session (completed by helpers).  Reflection notes (completed by helpers).  Telehealth experience survey containing validated measures on perceived usefulness, perceived ease of use, self-efficacy in using telehealth, and perceived difficulty of tasks (completed by participants).  Survey with questions about experience with helpers (completed by participants).  Semi-structured interviews about helping sessions (completed by participants).    Electronic Health Record (EHR) data to examine previous telehealth experience and if telehealth modality changed after session (completed by research analyst). | Of 239 patients, 34 (14.2%) completed the intervention.  Post-training, there was no significant difference between intervention and non-intervention groups on self-efficacy to use telehealth.  Post-training, there was no significant difference between intervention and non-intervention groups on perceived difficulty in using video visit software.  Post-training, intervention participants were significantly less satisfied with their video visit experience than non-intervention participant (*P* = .002).  Three training strategies were identified for cognitive load reduction: 1) providing step-by-step guidance for configuring and learning, 2) building rapport to create confidence with problem-solving, 3) being on the same page to counter informational distractions. |
| **Authors:**  Chu et al. 2022  **Article type:** Empirical | **Design:** Pre-post quality improvement (QI) intervention.  **Population and setting:** 1427 older adults (≥ 65 years), academic primary care clinic, USA. | To help older adults access video visits at an academic primary care practice. | **Delivery mode:** Phone.  **Frequency:** Single phone call.  **Trainer:** Research team member.  ***N* participants received training:** 192. | Number of patients called.  Outcomes of phone calls (what stage patients were at in relation to using video visits).  Final outcome of scheduled visit (cancelled, no-show completed: telephone, video, in-person). | 1427 patients were called, with 1025 (71.8%) patients reached.  Of the 1025 patients reached, 192 (18.7%) accepted technical assistance to video-enable their devices.  Of the 192 patients who accepted assistance, 149 (77.6%) were successfully video-enabled.  Of the 1025 patients reached, 40.4% completed their visit by video, 26.5% by telephone, and 1.4% by in-person visit, while 29.6% cancelled and 2.1% no-showed. |
| **Authors:**  Gulati et al. 2022  **Article type:** Empirical | **Design:** Post-programme evaluation.  **Population and setting:** 335 older adult patients (≥ 70 years), academic geriatrics primary care, USA | To support community-dwelling older adults by addressing barriers to health care delivery. | **Delivery mode:** Phone. Step-by-step instructions via email.  **Frequency:** Single phone call.  **Trainer:** Health care student volunteer.  ***N* participants received training:** 25. | Number of phone calls completed.  Number of video visits completed.  Web-based survey for post-programme evaluation (completed by student volunteers). | Of the 335 patients called, students successfully spoke with 247 patients (74%) and assisted 25 of 28 patients (89%) who requested telehealth training.  22 patients completed a video-based telehealth appointment within two months of training.  After participating in the training, all students (n=21) reported feeling comfortable communicating with patients or caregivers by phone, with 95% reporting confidence in relationship-centred communication. |
| **Authors:**  Gusdorf et al. 2021  **Article type:** Empirical | **Design:** Retrospective cohort study.  **Population and setting:** 45,803 adult patients, academic medical centre, USA. | To support patients’ access to telehealth. | **Delivery mode:** Phone. Step-by-step instructions via email.  **Frequency:** Single phone call.  **Trainer:** Medical student volunteer.  ***N* participants received training:** 5182. | Non-completion of any visit.    Completion of phone-only versus audio-visual telehealth visits. | Patients who received a pre-visit phone call had decreased rates of failed video-visits.  Those with non-commercial insurance and those of Black race were more likely to have a failed video visit. |
| **Authors:**  Hawley et al. 2020  **Article type:** Empirical | **Design:** Exploratory sequential mixed methods.  **Population and setting:** 50 Veterans attending a geriatric renal clinic, USA | To identify and address patient-perceived barriers to integrating home telehealth visits. | **Delivery mode:** Phone call. Video test call. Instructional guide.  **Frequency:** Single phone call and single video call.  **Trainer:** Telehealth technician.  ***N* participants received training:** 32. | In-person needs assessment (completed by participants).  Telephone post-visit interviews (completed by participants). | All participants who received training (n=32) successfully completed a telehealth visit.  The majority of interviewed participants (n=12) reported that the training and instructions were helpful (n=11, 92%).  Participants who required training spent an average of 30 minutes training for the visit, with an additional 15 to 30 minutes if they completed a test call. |
| **Authors:**  Hoffman et al. 2020  **Article type:** Brief communication | **Design:** Brief Communication.  **Population and setting:** Patients at an academic medical centre. | To help patients of all levels of technological literacy to become comfortable with telehealth system and troubleshooting technical issues. | **Delivery mode:** Phone call.  **Frequency**: Single phone call.  **Trainer:** Hospital staff & IT personnel.  ***N* participants received training:** Not specified. | Not specified. | Not specified. |
| **Authors:**  Jezewski et al. 2022  **Article type:** Empirical | **Design:** Pre-post intervention survey.  **Population and setting:** 630 older adults (80% > 65 years) in community settings, USA. | To reduce telehealth barriers in vulnerable people through education and improve knowledge of telehealth. | **Delivery mode:** PowerPoint presentation. Written guide.  **Frequency**: Single 20-minute presentation.  **Trainer:** Medical student & self-directed.  ***N* participants received training:** 630. | Pre-training survey with questions about demographic information, internet access, and familiarity with telehealth (completed by participants).  Post-training survey with questions on understanding of telehealth, willingness to use telehealth, and assistance needed for telehealth (completed by participants. | 257 participants completed surveys. Pre-training, 39% of participants were familiar with telehealth.  Post-training, 70% of participants understood how to access telehealth, and 39% said they would use telehealth in the future.  Post-training, a larger proportion of “in-person” (73%) learners were willing to use telehealth than “at-home” learners (41%) (*P* < .001). |
| **Authors:**  Meyer et al. 2021  **Article type:** Brief communication | **Design:** Brief communication.  **Population and setting:** Patients at an academic health centre, USA. | To facilitate the rapid expansion to telemedicine. | **Delivery mode:** Phone call. Written guide.  **Frequency:** Single phone call.  **Trainer:** Volunteer medical students. Care team.  ***N* participants received training:** Not specified. | Not specified. | Not specified. |
| **Authors:**  Neumann et al. 2023  **Article type:** Empirical | **Design:** Convergent parallel mixed methods. Pre-post design.  **Population and setting:** 43 older adults (≥ 65 years), community setting, USA. | To train older adults to engage with technology and online health-related activities. | **Delivery mode:** Video call. Booklet. Free iPad.  **Frequency:** Seven sessions over two months.  **Trainer:** Volunteers.  ***N* participants received training:** 31. | Pre-training survey with questions on demographic information, internet use, and confidence with telehealth (completed by participants).  Post-training survey with questions on telehealth confidence and skills (completed by participants).  Pre- and post- training semi-structured interviews (completed by participants). | Three months post-training, over 50% of participants reported needing no or little help with telehealth tasks.  A significant increase (*P* = .003) was found in the mean confidence level for video visits, from pre- to three months post- training.  Interviewed participants reported that learning was facilitated by self-pacing, repetition, and longitudinal support from volunteers. |
| **Authors:**  Pichan et al. 2021  **Article type:** Empirical | **Design:** Mixed methods. Post-programme evaluation.  **Population and setting:** 80 older adults (≥ 65 years), geriatrics clinic, USA. | To help geriatric patients get connected to telehealth. | **Delivery mode:** Phone call. Instructions.  **Frequency:** Three phone calls over one week.  **Trainer:** Medical students.  ***N* participants received training:** 71 | Proportion of completed virtual visits that were conducted by video versus telephone.  Post-programme survey with questions on positives and negatives of training (completed by providers and trainers).  Phone interviews (completed by participants). | Over a 10-week evaluation period, providers whose patients worked with the volunteers had a video visit rate of 43% compared to 19.2% prior to participation in the programme.  14 patients and 7 providers contributed feedback. The main theme was of appreciation for increased patient confidence with technology for telehealth. Patients highlighted the amount of time volunteers put into helping them. |
| **Authors:**  Smith et al. 2020  **Article type:** Brief communication | **Design:** Brief communication.  **Population and setting:** Patients in large specialty care clinic, USA. | To facilitate telemedicine implementation. | **Delivery mode:** Phone call. Download instructions.  **Frequency:** Single phone call.  **Trainer:** Nursing staff.  ***N* participants received training:** Not specified. | Not specified. | Not specified. |
| **Authors:** Spindler et al. 2021  **Article type:** Brief communication | **Design:** Brief communication.  **Population and setting:** Patients in a neurology department. | To rapidly implement teleneurology. | **Delivery mode:** Pre-recorded video.  **Frequency:** Single video.  **Trainer:** Self-directed.  ***N* participants received training:** Not specified. | Not specified. | Provides a list of tips for patients on preparing for a teleneurology visit. |
| **Authors:** Taylor et al. 2023  **Article type:** Empirical | **Design:** Exploratory sequential mixed methods.  **Population and setting:** 94 older adults (≥ 60 years), community setting, USA. | To increase telehealth accessibility among older adults by providing training to increase telehealth competency. | **Delivery mode:** Web-based modules containing videos and written guides.  **Frequency:** 5 modules.  **Trainer:** Self-directed.  ***N* participants received training:** 53 | Post-training qualitative interviews to evaluate acceptability and solicit recommendations (completed by participants).  Pre- and post- training competency survey “Telehealth Competency Questionnaire-Consumer” (completed by participants). | There was a significant improvement in perceived telehealth competency from pre- to post- training (*P* <.001).  Participants critiqued the initial training modules as having limited information on telehealth privacy and advised improving the accessibility of the modules design. |
